# Account of Diseases in an Eastern District of London, from the 20th of January, to the 20th of February, 1807

**Published:** 1807-03

**Authors:** 


*Account of Diseases in an Eastern District of London, from the 20th of January, to the 20th of February,* 1807.

| | | | |
|---|---|---|---|
| ACUTE DISEASES. | | Apoplexia - - | 1 |
| Pneumonia - - | 3 | Fluor Albus - - | 7 |
| Peripneumonia Notha | 4 | Amenorrhœa - - | 5 |
| Catarrhus - - | 3 | Palpitatio - - - | 2 |
| Typhus - - - | 1 | Hypochondriasis - | 5 |
| Rheumatismus Acutus | 3 | Hysteria - - - | 4 |
| CHRONIC DISEASES. | | Rheumatismus Chronicus | 25 |
| Tussis - - - | 19 | PUERPERAL DISEASES. | |
| Tussis cum Dyspnœa | 17 | Menorrhagia Lochialis | 4 |
| Phthisis Pulmonalis - | 3 | Dolores post partum - | 6 |
| Hæmoptoe - - | 2 | Mastodynia - - | 4 |
| Pleurodyne - - | 4 | INFANTILE DISEASES. | |
| Gastrodynia - - | 5 | Pertussis - - - | 5 |
| Ascites - - - | 3 | Aphthæ - - - | 6 |
| Cephalalgia - - | 4 | Ophthalmia Purulenta | 4 |
| Vertigo - - - | 2 | Vermes - - - | 3 |

The influence which the temperature of the atmosphere has on the diseases of the pulmonary system, has been remarkably exemplified in the present season. The uncommon mildness of the temperature has been particularly favourable to those patients who have an annual return of these complaints. The number of aged persons who have fallen a sacrifice to these diseases, during the present season, has been comparatively small. The few very warm days which we had, afforded temporary relief to those who were afflicted with catarrh, peripneumony, cough, or dyspnœa. This alleviation of symptoms, however, was very transient, and the sudden change which took place about the 17th or 18th day of the present month, produced in many instances, a very considerable aggravation of symptoms. These circumstances, which occur pretty uniformly to the notice of an accurate observer, point out the importance, and indeed the absolute necessity of an attention, in persons subject to these complaints, to the various means of guarding against their occasional cause. Avoiding every unnecessary exposure to the air, guarding the surface of the body by very warm clothing, or, when circumstances will admit of it, taking up their residence in those parts of the kingdom, where they may enjoy the most equable state of temperature, are precautions worthy the attention of the persons referred to.

